# The effect of mobile app-based self-care training on the quality of marital relations and the severity of menopausal symptoms in postmenopausal women: a clinical trial study in Iran

**DOI:** 10.1186/s12905-023-02463-4

**Published:** 2023-06-12

**Authors:** Foozieh Rafati, Najme Pourshahrokhi, Raziyeh Sadat Bahador, Neda Dastyar, Akbar Mehralizadeh

**Affiliations:** 1 School of Nursing and Midwifery, Jiroft University of Medical Sciences, Jiroft, Iran; 2 Medical Informatics, Kerman University of Medical Sciences, Kerman, Iran; 3 Department of Midwifery, School of Nursing and Midwifery, Jiroft University of Medical Sciences, Jiroft, Iran; 4 School of Medicine, Jiroft University of Medical Sciences, Jiroft, Iran

**Keywords:** Self-care, Menopause, Training, Smartphone application

## Abstract

**Background:**

Educational interventions for self-care are a necessary solution to help postmenopausal women properly deal with menopausal problems. The present study aimed to investigate the effect of self-care training using an application on the quality of marital relations and the severity of menopausal symptoms in postmenopausal women in Iran.

**Methods:**

In this study, 60 postmenopausal women selected using the convenience sampling method were divided into two groups, intervention and control, using simple random allocation (lottery). The intervention group used the menopause self-care application for eight weeks in addition to routine care, but the control group only received the routine care. The Menopause Rating Scale (MRS) and the Perceived Relationship Quality Components (PRQC) questionnaire were completed in two stages, before and immediately after eight weeks, in both groups. Data were analyzed using SPSS software (version 16), descriptive (mean and standard deviation), and inferential (ANCOVA and Bonferroni post hoc) statistics.

**Results:**

The ANCOVA results showed that the use of the menopause self-care application decreased the severity of the participants’ menopause symptoms (*P* = 0.001) and improved the quality of their marital relations (*P* = 0.001).

**Conclusion:**

Implementation of a self-care training program through the application helped improve the quality of marital relations and reduce the severity of postmenopausal women's symptoms, so it can be used as an effective method to prevent the unpleasant consequences of menopause.

**Trial registration:**

The present study was registered at https://fa.irct.ir/ on 2021–05-28 (registration number: IRCT20201226049833N1).

## Background

Menopause is a biological stage in a woman's life that is diagnosed after 12 months of permanent cessation of menstruation [1]. Menopause usually occurs between the ages of 45 and 55 and is known as a transition from one stage to another in a woman's life [2]. During this period, most women experience different changes [3]. These changes can be social, physical, and psychological [4]. Most postmenopausal women face common issues such as hot flashes, headaches, mental and physical exhaustion, sleep problems, mood changes (anxiety, depression, irritability, and stress), joint and muscle pain, osteoporosis, heart disease, bladder problems, and sexual problems [5, 6]. Some women may experience one or two mild symptoms while others may have more severe and distressing symptoms [7]. Studies have shown that hot flashes and night sweats are the main symptoms, and approximately 75% of women experience vasomotor symptoms (VMS) around the time of menopause [8]. Moderate and severe VMS causes a decrease in mood, productivity, and concentration and can significantly affect the quality of life of women [9].

Nowadays, quality of life is accepted as a multidimensional concept [10]. The quality of marital relations, which may be disturbed during menopause, is one of the dimensions of quality of life [11]. The quality of marital relations includes various dimensions of a couple's relationship, such as sexual relations, compatibility, happiness, life satisfaction, cohesion, and commitment [12–14]. Sexual relations are among the most important aspects of marital relations that are affected during menopause [3]. One of the problems faced by women during this period is pelvic floor muscle relaxation, vaginal dryness, urinary problems, painful intercourse, sexual dysfunction, and, consequently, sexual dissatisfaction [15]. These changes, however, can be adjusted and controlled using interventions such as education and technology-based interventions, leading to an improvement in sexual performance [5, 16, 17].

Studies have shown that due to the lack of knowledge about menopause, awareness-raising and provision of knowledge to women are necessary [18]. Since women spend on average one-third of their lives in the postmenopausal period, it is very important to pay attention to their health status during this period [19]. Nurses and midwives are considered vital components of the health care team since the nature of their everyday work is such that it provides an opportunity to encourage and influence postmenopausal women to acquire correct self-care behaviors [20, 21]. Educational interventions to teach the correct principles of self-care are a necessary solution to improve health and help postmenopausal women better cope with menopause problems and personal disabilities and increase self-efficacy [14]. Like increasing awareness and modifying lifestyle, self-care can also help solve health-related problems [6]. According to the Orem model, self-care is a learned behavior that can meet the numerous needs of patients during diseases or once health deviations occur. According to this theory, humans can take care of themselves, and when this ability is compromised, the caregiver can enable them to recover their self-care ability by providing direct and compensatory care or educational-supportive care [22]. Therefore, the necessity of improving self-care ability through education is undeniable [23].

In today's world, many aspects of daily life have changed due to the integration of information and communication technology. In this regard, the use of modern methods, such as self-care training through applications, has received better reception due to its ease of use and time effectiveness [24]. On the other hand, postmenopausal women are among the fastest-growing population groups in the world, and virtual interventions have been welcomed as alternative ways for postmenopausal women to manage and cope with their symptoms [25]. The development of the mobile health (mHealth) platform to support women going through menopause is increasing around the world [7]; previous studies have confirmed the impact of mhealth on self-efficacy, quality of life, the severity of menopausal symptoms, and physical symptoms [25–28]. Therefore, it seems very necessary to design mHealth-based programs in the national language to manage the symptoms of postmenopausal women. Despite the great importance of the topic, as far as we know, no published data has been conducted to investigate the effect of menopausal self-care training through an application on the quality of marital relationships and the severity of menopausal symptoms of postmenopausal women in Iran. Therefore, this study hypothesized that mobile app-based self-care training improves the quality of marital relations and declines the severity of menopausal symptoms in postmenopausal women. We hope that the results of this study can take a useful and constructive step towards improving the self-care behaviors of postmenopausal women in Iran and other parts of the world.

## Methods

### Study design

**Fig. 1:**
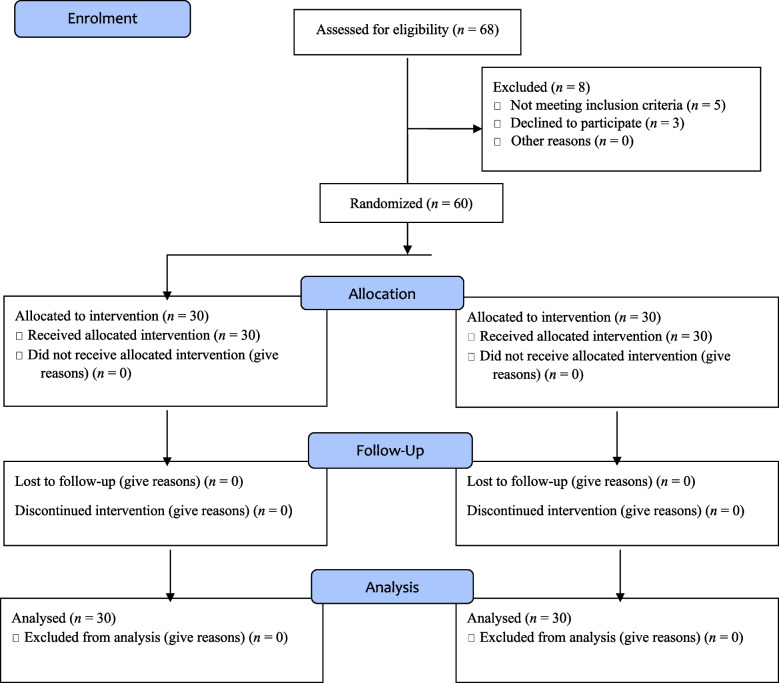
CONSORT flow diagram of the randomized controlled trial

This randomized, controlled, single-blind trial was conducted from the beginning of August to the end of November 2021 on 60 postmenopausal women who were referred to Jiroft’s comprehensive health service centers in the south of Iran. All parts of the study were conducted according to the Consolidated Standards of Reporting Trials (CONSORT) statement [29] (Fig. 1). This trial was recorded in the Iranian registry of clinical trials (registration date: 2021–05-28, registration number: IRCT20201226049833N1).

### Research environment and participants

The study population consisted of all postmenopausal women aged 45 to 65 years who were referred to Jiroft’s comprehensive health service centers to receive routine menopause care (Such as control of vital signs and blood sugar, breast examination, Pap smear test and weighing) and met the inclusion criteria.

Women who provided informed consent to participate in the study, were aged 45 to 65 years, had complete cessation of menstruation in the last 12 months, had natural menopause (not with the use of drugs, hysterectomy, or oophorectomy), were in the second or third year of menopause, were literate, had access to an Android smartphone, had experience using other mobile applications, did not suffer from or have a history of serious and chronic physical and mental illness, were not addicted to cigarettes or drugs, had not experienced any unfortunate incidents in life in the last 6 months (Such as: death of close relatives), were not receiving hormone replacement therapy, did not use herbal medicines, and had not participated in similar research projects in the last 6 months were included in the study. These criteria were evaluated using a checklist.

The exclusion criteria included unwillingness to continue participating in the study, not using the application during the intervention, occurrence of any mental crisis and severe physical illness during the study, and failure to complete all questionnaire items.

The sample size was estimated to be 24 subjects per group based on the mean and standard deviation of the menopausal symptom severity score in a study by Shakiba et al. [30] with a 95% confidence interval and 90% statistical test power and based on the following formula. In this study by taking 10% possible dropout into account and to increase confidence in the results, 30 subjects in each group and a total of 60 subjects were estimated as sample size.

$$n=\frac{{\left({S}_{1}^{2}+{S}_{2}^{2}\right) \left({Z}_{1-{\alpha }_{2}}+{Z}_{1-\beta }\right)}^{2}}{{d}^{2}}$$

$$\begin{array}{ccc}X_1=19.37&X_2=24.9&\begin{array}{ccc}S_1=4.93&S_2=6.67&\begin{array}{cc}Z_1-\beta=1.28&Z_1-\frac\alpha2=1.96\end{array}\end{array}\end{array}$$

### Sampling and randomization

In the present research, the convenience sampling method was used. First, three urban comprehensive health service centers were selected using a lottery among Jiroft's comprehensive health service centers. The main researcher prepared an initial list of all postmenopausal women covered by 3 centers, contacted them by phone, evaluated them considering the inclusion criteria, and prepared a final list of eligible postmenopausal women. Then, a third person who did not participate in the research protocol divided the subjects of the final list into two groups, control, and intervention, by convenience random (lottery) method.

### Application design

**Fig. 2:**
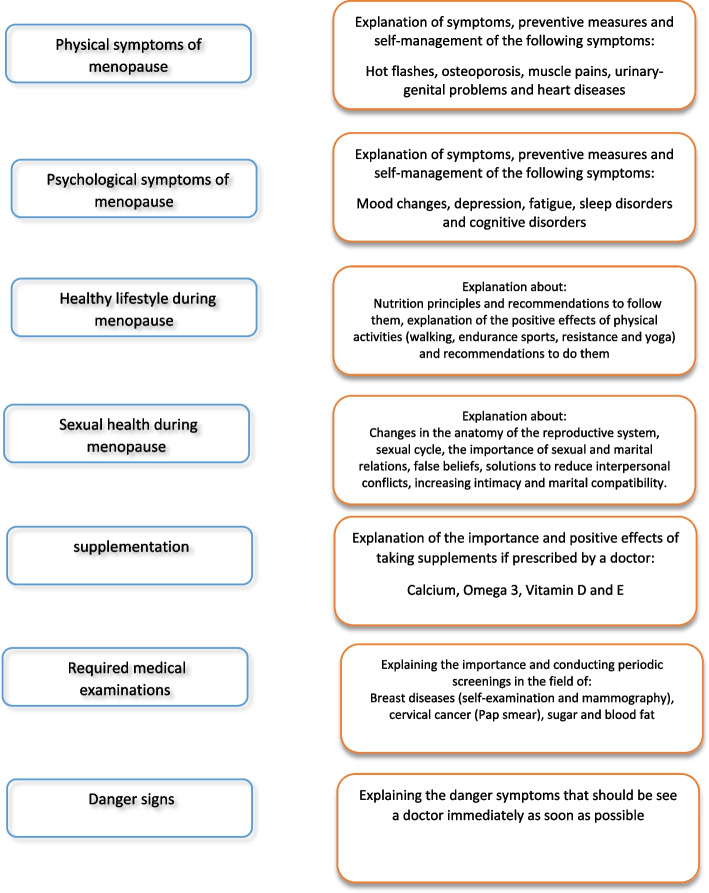
The main menu of the menopause self-care application

The menopause self-care application was a simple Persian-language application that was developed to provide a wide range of information about menopause and menopausal symptom management training based on the Analysis, Design, Development, Implementation, and Evaluation (ADDIE) model [31]. The review of existing and valid texts on menopause and interviews with seven postmenopausal women, four gynecologists, three midwifery experts, and a nutritionist were used to design and compile the content of the application. Educational requirements of postmenopausal women, issues, problems of the menopause period, and their self-management solutions were determined, and the main menu of the application was created (Fig. 2). In the analysis and design phase, the first version of the menopause self-care application was developed by a professional web developer. This application was designed for the Android operating system with JAVA programming language in Android Studio version 3.1.4 and included a training and information module. SQLite website was used to design and install the database, and DB Browser for SQLite version 3.10.1 was used to transfer the data to the database.

The initial version was assessed by a team consisting of gynecologists, nutritionists, senior obstetricians, and health information technology experts. Also, the application was used for one week by five postmenopausal women who were not included in the study to check its usability. The opinions of the team of postmenopausal women and experts were collected and several minor modifications were made to the application, and the final version of the *Menopause Self-Care* application was developed. Then, the effectiveness of its use on the severity of menopausal symptoms and the quality of marital relationships of postmenopausal women were evaluated.

### Education and information module

This module included information about physical and mental-psychological issues and their self-management solutions, healthy lifestyle, sexual health, nutrition, supplements, required medical examinations, and warning signs during the menopause period. The information was placed in the application in the form of multimedia content (text, image, sound, and video) in simple language.

### Outcome measurement and data collection instruments

The data collection tool was a questionnaire consisting of three parts completed in the form of a self-report: 1- Demographic information checklist, 2-main outcomes: A- Menopause Rating Scale (MSR), and B- Perceived Relationship Quality Components (PRQC) questionnaire.

1- The demographic information checklist assessed age, number of pregnancies, abortions, births, income, education level, and occupation.

A- Menopause Rating Scale (MSR): This is a valuable international tool for assessing the severity of menopause symptoms [32] examining 11 symptoms related to menopause. The answers to these questions are on a 5-point Likert scale from (not at all) to (very strong). The minimum score for each question is 0 and the maximum score is 4, and the total score range is 0 to 44. The lower the overall MRS score, the less severe the menopausal symptoms experienced. The validity and reliability of this questionnaire have been confirmed in Iran [30, 33]. In the current study, the reliability of this tool was checked through Cronbach's alpha and 0.81 was obtained for the entire scale.

B- Perceived Relationship Quality Components (PRQC) questionnaire: This questionnaire was designed in England by Fletcher et al. (2000) [34] and contains 18 questions. The answers to these questions are on a 7-point Likert scale from “not at all” to “completely.” The minimum score for each question is 1 and the maximum score is 7, and the total score range is 18 to 126. Lower scores indicate a low level and higher scores indicate a high level of marital relationship quality. The validity and reliability of this questionnaire have been confirmed in Iran [35, 36]. In the present study, the reliability of this tool was confirmed by a Cronbach's alpha of 0.88 for the entire scale.

### Data collection

After developing the final version of the application, the researcher contacted all the subjects in the intervention and control groups and invited them to one of the comprehensive health service centers. The objectives, the method of conducting the research, and the voluntary participation in the study were explained to the subjects, and written informed consent was taken. In the same stage, the pre-intervention data was gathered through the completion of the demographic information checklist, MRS, and PRQC by both groups. After Eight weeks, the control group (who received only routine care) was invited to come to one of the comprehensive health service centers for a follow-up assessment. (At first, data was collected from the control group to minimize the transfer of information between the two groups, from August 1, 2021, to September 30, 2021).

The educational intervention started after data collection from the control group (from October 1, 2021, to November 30, 2021). Then the intervention group was invited to one of the comprehensive health service centers. The main researcher installed the menopause self-care application on their smartphones. Using the application was taught to them in person. They were asked to use the menopause self-care application for eight weeks to receive the necessary information about self-management of menopausal symptoms. One week after installing the application, the women in the intervention group were contacted, their questions were answered, and their problems regarding how to use the application were addressed. One of the researchers contacted participants daily for answering their questions and fixing their problems regarding how to use the application. Then, twice a week, the first and responsible author reminded the intervention group about the use of the application by text message. In addition, the phone number of one of the researchers (ND) was given to participants so that they could call if they had any questions or problems. Eight weeks after the beginning of the educational intervention, post-intervention data was gathered from the intervention group. The intervention duration (eight weeks) was selected based on previous studies [37, 38].

### Statistical analysis

The data were analyzed using SPSS software version 16 (IBM, Armonk, New York, Version 16). After data collection, data entry was done on the dataset designed by N D. The data entry accuracy and screening were done under the supervision of F R and A MAZ. The normal distribution of the quantitative variables was first confirmed using the Kolmogorov–Smirnov test. Then the frequency, percentage, mean, standard deviation, minimum, and maximum were determined using descriptive statistics. Then, the independent *t*-test was used to compare the mean score of the intervention and control groups, the chi-square test was also used to compare the frequency of the qualitative variables in both groups and analysis of the covariance test (ANCOVA) and post hoc Bonferroni test was used to determine the effectiveness of the menopause self-care application with the simultaneous control of some confounding variables. The significance level in this study was *P* < 0.05.

## Results

The average age of the women was 53.57 ± 3.41 and 53.93 ± 3.45 in the intervention and control groups respectively. The education level of most of the participants, 48 subjects (80%), was a high school diploma or lower. Twenty-three subjects (38.3%) were homemakers, and 40 subjects (66.6%) had an average or higher income. All research subjects had a husband and lived with their husband at the time of the study. Other demographic variables are reported in Table 1. According to the results of the independent *t*-test, there was no significant difference between the intervention and control groups in terms of average age, number of pregnancies, number of childbirths, and number of abortions (*P* > 0.05). According to the results of the chi-square test, no statistically significant difference was observed between the intervention and control groups in terms of occupation, income, and education level (*P* > 0.05) (Table 1).

**Table 1:** Demographic characteristics (quantitative and qualitative) of postmenopausal women in both control and intervention groups

Demographics Quantitative variable		Control	intervention	*P*-value
		Mean (standard deviation)	Mean (standard deviation)	
**Age**		53.93 ± 3.45	53.57 ± 3.41	0.68*
**Number of pregnancies**		4.27 ± 1.51	4.03 ± 1.45	0.54*
**Number of abortions**		0.8 ± 1.03	0.97 ± 1.12	0.55*
**Number of childbirths**		3.50 ± 1.01	3.10 ± 0.92	0.11*
**Demographics** **Quantitative variables**		Control	Intervention	***P*-value**
		Number (Percentage)	Number (Percentage)	
**Income**	Low	8 (26.67)	12 (40)	0.44**
	Medium	12 (40)	8 (26.67)	
	Good	10 (33.33)	10 (33.33)	
**Education level**	High school diploma and below	12 (40)	10 (33.33)	0.56**
	Associate degree	11 (36.67)	15 (50)	
	Bachelor's degree and higher	7 (23.33)	8 (16.67)	
**Occupation**	Homemaker	12 (40)	11 (36.66)	0.94**
	Retired	11 (36.66)	11 (36.66)	
	Self-employed	7 (23.34)	8 (26.68)	

^*^Independent *t,* ** chi-Square

The results showed that the mean score of the severity of menopause symptoms for the intervention group in the post-intervention stage was 30.27 ± 6.09, which was lower than the mean score of the control group 39.10 ± 3.71. The mean score of the severity of menopause symptoms in the post-intervention stage of the intervention group was 30.27 ± 6.09, which was lower than the pre-intervention mean score, of 36.63 ± 5.27.

The mean score of the quality of marital relations in the post-intervention stage for the intervention group was 82.90 ± 16.06, which was higher than the mean score for the control group, 71.20 ± 16.16. The mean score of the quality of marital relations in the post-intervention stage for the intervention group was 82.90 ± 16.06, which was higher than the pre-intervention mean score, 76.87 ± 15.68 (Table 2).

**Table 2:** The mean score of severity of menopausal symptoms and quality of marital relationships of menopausal women in the control and intervention groups

Variable	Mean (standard deviation)		Mean (standard deviation)	
	Pre-intervention		post-intervention	
	Control	intervention	Control	intervention
**The severity of menopause symptoms**	36.33 ± 5.40	36.63 ± 5.27	39.10 ± 3.71	30.27 ± 6.09
**Quality of marital relations**	76.87 ± 18.32	76.87 ± 15.68	71.20 ± 16.16	82.90 ± 16.06

The results of the difference test between groups using covariance analysis showed that the use of the menopausal self-care application had a significant effect on the severity of menopausal symptoms of menopausal women and the quality of their marital relationships (*P* < 0.001) (Table 3).

**Table 3:** The results of the comparison between the groups using analysis of covariance for the variables of the severity of menopause symptoms and quality of relationships

Variable	Source	The sum of the squares	Degrees of freedom	Mean square	*F*	*P*-value	Partial Eta squared
**The severity of menopause symptoms**	Model	3115.21	3	1038.40	46.30	0.001	0.713
	Pre-intervention	1924.33	1	1924.33	85.814	0.001	0.605
	group	1170.41	1	1170.41	52.19	0.001	0.482
	Pre-intervention*group	20.45	1	20.45	0.912	0.344	0.016
	Error	1255.77	56	22.42	-	-	-
	Total	89,573.00	60	-	-	-	-
**Quality of marital relations**	Model	14,211.71	3	4737.23	91.44	0.001	0.830
	pre-intervention	12,071.10	1	12,071.10	233.00	0.001	0.806
	group	2053.35	1	2053.35	39.63	0.001	0.414
	pre-intervention*group	87.25	1	87.25	1.68	0.20	0.029
	Error	2901.13	56	51.80	-	-	-
	Total	373,315.00	60	-	-	-	-

R Squared for Severity of menopause symptoms = 0.71 (Adjuster R Squared = 0.69),

R Squared for Quality of marital relations = 0.83 (Adjuster R Squared = 0.82)

According to the results of the Bonferroni test (Table 4), the difference in the mean score of the intensity of menopausal symptoms (intervention/control) was positive, so the mean score of the intensity of menopausal symptoms for the intervention group was significantly lower than that of the control group (*P* < 0.01). Therefore, the use of the self-care application during menopause reduced the severity of menopausal symptoms in menopausal women. The Bonferroni test results also showed that the difference in the mean scores of marital relationship quality (intervention/control) was negative, so the mean score of the quality of marital relationships of the intervention group was significantly higher than that of the control group (*P* < 0.01). Therefore, the use of the menopause self-care application improved the quality of marital relationships of menopausal women.

**Table 4:** The results of the comparison of the mean scores adjusted by the Bonferroni test for the variables of the intensity of menopause symptoms and quality of marital relations

Variable	Group (i)	Group (j)	Mean difference (i-j)	Standard deviation	*P*-value	95% confidence interval for the difference in means	
						Lower bound	Upper bound
**The severity of menopause symptoms**	Control	Intervention	9.06	1.22	0.001	6.61	11.51
**Quality of marital relations**	control	Intervention	-11.70	1.85	0.001	-15.44	-7.97

## Discussion

The results of the present study showed that the use of the self-care mobile app for eight weeks reduced the symptoms of menopause in the intervention group compared to the control group. This result is in line with the results of previous studies. For example, Park et al. (2022) [39] showed that the use of a mobile self-care program reduced menopausal symptoms and improved the self-efficacy and quality of life of women who underwent menopause following chemotherapy. The results of a study by Eun-Ok et al. (2017) [27] also showed that a web-based program to promote physical activity reduced menopausal symptoms in women. In another study, it was reported that lifestyle management training through Facebook improved the quality of life and health habits, sleep quality, and physical activity pattern of postmenopausal women [40]. Another study in Iran confirmed the effect of virtual programs on improving the quality of life, vasomotor symptoms, and physical and sexual aspects of women [26]. Although Eun-Ok et al. (2017) [27] believe that these effects may be influenced by the Hawthorn effect, other studies that reported the improving effect of mHeath and virtual programs on objective outcomes, such as waist circumference, weight, blood pressure, serum lipid levels and pain [25, 28], indicate that the effect of mHeath on health-related outcomes is more real than can be justified by the Hawthorn phenomenon.

The results of the present study also showed that the use of a self-care mobile app for eight weeks in postmenopausal women improved the quality of marital relationships of postmenopausal women in the intervention group compared to the control group. The results of the study by Kashfi et al. (2021) [26] showed that an educational program on the WhatsApp platform improved the quality of life of postmenopausal women. However, a review study reported that the results of studies on the effect of virtual programs on sexual performance were contradictory [25]. Therefore, it is suggested that studies with a large sample size be repeated so that the impact of mhealth interventions on sexual performance and the quality of marital relationships can be assessed with more certainty. The use of self-care and self-management methods may directly improve the symptoms related to the genital tract after menopause (such as vaginal dryness) or indirectly improve marital relations by reducing the severity of menopause symptoms. The severity of menopause symptoms has a direct relationship with the quality of marital relations [14].

Women spend more than one-third of their lives after menopause [19], and the severity of menopause symptoms and the quality of marital relationships affect the quality of life of their lives [14]. Therefore, they must be empowered for self-care. Since today the development of mHeath platforms to support women during menopause is increasing worldwide and women express their desire to use mobile apps to manage their menopause symptoms [7], researchers and health workers should pay attention to the design of self-care apps for postmenopausal women in the national language of each country. Given that mobile applications are attractive to people, including postmenopausal women, due to their affordability, usability at any time and place, and high quality, they can provide an opportunity for teaching self-care and implementing support programs in an innovative, efficient, and cost-effective way.

Failure to control the Hawthorne effect, inability to generalize the results to other communities and not measuring the durability of the results in the long term were the limitations of this research. The data were also collected in the form of self-report, which means that the researcher was unable to confirm their accuracy and had to trust the accuracy of the statements of the individuals. It is suggested that future research be conducted using other platforms of virtual education, for different populations and longer follow-up periods and to determine its real long-term effect.

## Conclusion

The results showed that the implementation of the self-care training program using the mobile-based application helped improve the quality of marital relationships and reduce the severity of menopausal symptoms of menopausal women; therefore, implementing this training method along with routine care may be effective for postmenopausal women who cannot participate in face-to-face training sessions due to physical problems, distance, cost, etc.

## Data Availability

The datasets used and/or analyzed during the current study are available from the corresponding authors upon reasonable request.

## References

[1] Gebretatyos H, Ghirmai L, Amanuel S, Gebreyohannes G, Tsighe Z, Tesfamariam EH (2020). Effect of health education on knowledge and attitude of menopause among middle-age teachers. BMC Women's Health.

[2] Wang M, Gong W-W, Hu R-Y, Wang H, Guo Y, Bian Z (2018). Age at natural menopause and associated factors in adult women: findings from the China Kadoorie Biobank study in Zhejiang rural area. PloS one.

[3] Chaplin S (2016). NICE guideline: diagnosis and management of the menopause. Prescriber.

[4] Fan J, Yu C, Pang Y, Guo Y, Pei P, Sun Z (2021). Adherence to healthy lifestyle and attenuation of biological aging in middle-aged and older Chinese adults. The Journals of Gerontology: Series A.

[5] Hybholt M. Psychological and social health outcomes of physical activity around menopause: a scoping review of research. Maturitas. 2022:88–97. 10.1016/j.maturitas.2022.07.014. 10.1016/j.maturitas.2022.07.014 35964395

[6] Shepherd-Banigan M, Goldstein K, Coeytaux R, McDuffie J, Goode A, Kosinski A (2017). Improving vasomotor symptoms; psychological symptoms; and health-related quality of life in peri-or post-menopausal women through yoga: an umbrella systematic review and meta-analysis. Complement Ther Med.

[7] Osman A, Ahmad Noraimi NEI, Abdul Wahab N (2021). Healthy management of menopause: exploring issues and user requirements for mobile intervention. J Comput Res Innov (JCRINN).

[8] Formoso G, Perrone E, Maltoni S, Balduzzi S, Wilkinson J, Basevi V (2016). Short-term and long-term effects of tibolone in postmenopausal women. Cochrane Database Syst Rev.

[9] Monterrosa-Castro A, Romero-Pérez I, Marrugo-Flórez M, Fernández-Alonso AM, Chedraui P, Pérez-López FR (2012). Quality of life in a large cohort of mid-aged Colombian women assessed using the Cervantes Scale. Menopause.

[10] Thieken F, Timmermann L, Sohrabi K, Woopen C, Schmitz-Luhn B, Janhsen A (2022). Development of a multidimensional assessment tool for the evaluation of holistic quality of life in Parkinson’s disease. J Parkinson's Dis.

[11] Roesch AK, Warren A, Hill E (2021). The relationship between menopause and marital satisfaction in adult women. J Grad Educ Res.

[12] Tavakol Z, Nikbakht Nasrabadi A, Behboodi Moghadam Z, Salehiniya H, Rezaei E (2017). A review of the factors associated with marital satisfaction. Galen Med J.

[13] Sedaghatkhah A, BehzadiPoor S (2017). Predictingthe quality of marital relationship on the base of relationship beliefs, mindfulness and psychological flexibility. Quart Women Soc.

[14] Karimi L, Mokhtari Seghaleh M, Khalili R, Vahedian-Azimi A (2022). The effect of self-care education program on the severity of menopause symptoms and marital satisfaction in postmenopausal women: a randomized controlled clinical trial. BMC Womens Health.

[15] Trento SRSS, Madeiro A, Rufino AC (2021). Sexual function and associated factors in postmenopausal women. Rev Bras Ginecol Obstet.

[16] Keawtep P, Wichayanrat W, Boripuntakul S, Chattipakorn SC, Sungkarat S (2022). Cognitive benefits of physical exercise, physical-cognitive training, and technology-based intervention in obese individuals with and without postmenopausal condition: a narrative review. Int J Environ Res Public Health.

[17] Franco MM, Pena CC, de Freitas LM, Antônio FI, Lara LA, Ferreira CHJ (2021). Pelvic floor muscle training effect in sexual function in postmenopausal women: a randomized controlled trial. J Sex Med.

[18] Noroozi E, Dolatabadi NK, Eslami AA, Hassanzadeh A, Davari S (2013). Knowledge and attitude toward menopause phenomenon among women aged 40–45 years. J Educ Health Promot.

[19] Malik E, Sheoran P, Siddiqui A (2018). Health-promoting behaviors and menopausal symptoms: an interventional study in rural India. J Mid-life Health.

[20] Oluwatosin O (2012). Primary health care nurses’ knowledge practice and client teaching of early detection measures of breast cancer in Ibadan. BMC Nurs.

[21] Sasanpour M, Azh N, Alipour M. The effect of a midwife-based group discussion education on sexual dysfunction beliefs in rural postmenopausal women. Int J Women's Health. 2020:393–7. 10.2147/ijwh.s242621 10.2147/IJWH.S242621 PMC7213863 32440233

[22] Valizadeh S, Soheili A, Moghbeli G, Aliafsari E (2017). Applicablity of orem’s self-care model in Iran: an integrated review. Nurs Midwifery J.

[23] Hossein Mirzaee Beni Z, Maasoumi R, Pashaeypoor S, Haghani S (2022). The effects of self-care education based on the health literacy index on self-care and quality of life among menopausal women: a randomized clinical trial. BMC Women's Health.

[24] Mortara A, Vaira L, Palmieri V, Iacoviello M, Battistoni I, Iacovoni A (2020). Would you prescribe mobile health apps for heart failure self-care? An integrated review of commercially available mobile technology for heart failure patients. Card Fail Rev.

[25] Zou P, D'Souza D, Luo Y, Sun W, Zhang H, Yang Y (2022). Potential effects of virtual interventions for menopause management: a systematic review. Menopause.

[26] Kashfi SM, Rakhshani T, Farhoodi S, Motlagh Z, Bagherzadeh R, Kohan N (2021). The effect of education of physical activity via social networks on the quality of life in menopausal women: a randomized controlled trial. J Health Sci Surveill Syst.

[27] Im E-O, Kim S, Ji X, Park S, Chee E, Chee W (2017). Improving menopausal symptoms through promoting physical activity: a pilot Web-based intervention study among Asian Americans. Menopause.

[28] Park M-J, Kim H-S (2012). Evaluation of mobile phone and Internet intervention on waist circumference and blood pressure in post-menopausal women with abdominal obesity. Int J Med Informatics.

[29] Turner L, Shamseer L, Altman DG, Weeks L, Peters J, Kober T (2012). Consolidated standards of reporting trials (CONSORT) and the completeness of reporting of randomised controlled trials (RCTs) published in medical journals. Cochrane Database Syst Rev.

[30] Shakiba M, Rouhbakhsh M, Kermansaravi F, Navidian A. The effect of couple counseling on severity of menopausal symptoms in women. J Hayat. 2019;25(1):25–38. http://hayat.tums.ac.ir/article-1-2816-en.html. [In Persian].

[31] Seels BB, Richey RC. Instructional technology: The definition and domains of the field. Washington DC: Association for Educational Communications and Technology; 1994.

[32] Schneider H, Heinemann L, Rosemeier H-P, Potthoff P, Behre H (2000). The Menopause Rating Scale (MRS): reliability of scores of menopausal complaints. Climacteric.

[33] Jalili L, Yazdi Zadeh H, Sharifi N, Abedi P, Najar S, Asad Mobini E. The relationship between physical activity and the severity of menopause symptoms in menopausal women in Ahvaz, Iran. Iran J Obstet Gynecol Infertil. 2014;17(98):15–23. http://ijogi.mums.ac.ir/article_2830.html. [In Persian].

[34] Fletcher GJ, Simpson JA, Thomas G (2000). The measurement of perceived relationship quality components: a confirmatory factor analytic approach. Pers Soc Psychol Bull.

[35] Navidian A, Navabi Rigi S, Imani M, Soltani P. The effect of sex education on the marital relationship quality of pregnant women. J Hayat. 2016;22(2):115–27. http://hayat.tums.ac.ir/article-1-1424-en.html. [In Persian].

[36] Dastyar N, Sarasyabi AS, Shakiba M, Navidian A (2019). Impact of group assertiveness-based sexual training on the quality of marital relationships among female university students. J Educ Health Promotion.

[37] Rathnayake N, Alwis G, Lenora J, Mampitiya I, Lekamwasam S. Effect of health-promoting lifestyle modification education on knowledge, attitude, and quality of life of postmenopausal women. BioMed Res Int. 2020;2020:1–11. 10.1155/2020/3572903. 10.1155/2020/3572903 PMC7256760 32550229

[38] Duman M, Timur TS (2018). The effect of sleep hygiene education and relaxation exercises on insomnia among postmenopausal women: a randomized clinical trial. Int J Nurs Pract.

[39] Park JH, Jung YS, Kim JY, Bae SH (2022). Mobile web-based self-management program for breast cancer patients with chemotherapy-induced amenorrhoea: a quasi-experimental study. Nurs Open.

[40] Essner D (2019). Improving quality of life in menopausal women through lifestyle management: a web-based health promotion project: University of Alabama in HuntsvilleUniversity of Alabam.

[41] Association WM (2013). World Medical Association Declaration of Helsinki: ethical principles for medical research involving human subjects. JAMA.

